# Pain Research Forum: application of scientific social media frameworks in neuroscience

**DOI:** 10.3389/fninf.2014.00021

**Published:** 2014-03-11

**Authors:** Sudeshna Das, Patricia G. McCaffrey, Megan W. T. Talkington, Neil A. Andrews, Stéphane Corlosquet, Adrian J. Ivinson, Tim Clark

**Affiliations:** ^1^MassGeneral Institute for Neurodegenerative Disease, Massachusetts General HospitalCambridge, MA, USA; ^2^Department of Neurology, Harvard Medical SchoolBoston, MA, USA; ^3^Harvard NeuroDiscovery Center, Harvard Medical SchoolBoston, MA, USA; ^4^School of Computer Science, University of ManchesterManchester, UK

**Keywords:** social media, neuropathic pain, content management systems, Drupal

## Abstract

**Background:** Social media has the potential to accelerate the pace of biomedical research through online collaboration, discussions, and faster sharing of information. Focused web-based scientific social collaboratories such as the Alzheimer Research Forum have been successful in engaging scientists in open discussions of the latest research and identifying gaps in knowledge. However, until recently, tools to rapidly create such communities and provide high-bandwidth information exchange between collaboratories in related fields did not exist.

**Methods:** We have addressed this need by constructing a reusable framework to build online biomedical communities, based on Drupal, an open-source content management system. The framework incorporates elements of Semantic Web technology combined with social media. Here we present, as an exemplar of a web community built on our framework, the Pain Research Forum (PRF) (http://painresearchforum.org). PRF is a community of chronic pain researchers, established with the goal of fostering collaboration and communication among pain researchers.

**Results:** Launched in 2011, PRF has over 1300 registered members with permission to submit content. It currently hosts over 150 topical news articles on research; more than 30 active or archived forum discussions and journal club features; a webinar series; an editor-curated weekly updated listing of relevant papers; and several other resources for the pain research community. All content is licensed for reuse under a Creative Commons license; the software is freely available. The framework was reused to develop other sites, notably the Multiple Sclerosis Discovery Forum (http://msdiscovery.org) and StemBook (http://stembook.org).

**Discussion:** Web-based collaboratories are a crucial integrative tool supporting rapid information transmission and translation in several important research areas. In this article, we discuss the success factors, lessons learned, and ongoing challenges in using PRF as a driving force to develop tools for online collaboration in neuroscience. We also indicate ways these tools can be applied to other areas and uses.

## Introduction

Biomedical scientists rely heavily on the World Wide Web and Internet to do research and to perform literature, database, and information searches. Researchers are also increasingly adopting the Web to collaborate and exchange ideas. Web-based communities that bring together scientists from different disciplines, institutions, and sectors are called “collaboratories” (National Research Council, 1993). Collaboratories with embedded social media tools can increase the pace and quality of scientific collaboration with rapid, open and structured communication (Kouzes et al., 1996; Finholt and Olson, 1997).

However, several challenges for effective collaboration exist with respect to trust, independence, attribution, and intellectual property (Bos et al., 2007; Clark and Kinoshita, 2007). Scientists may prefer to work independently and may be restricted by the boundaries of institutions to freely exchange ideas. Moreover, there is the added complexity of communications across disciplines. Alzforum (http://www.alzforum.org) (Kinoshita and Clark, 2007)—a community of Alzheimer's disease researchers—was successfully able to overcome these challenges with a combination of high quality articles, neutrality, inclusiveness and editorial solicitation/moderation to gain trust and participation (Clark and Kinoshita, 2007). The Schizophrenia Research Forum (SRF) (http://www.schizophreniaforum.org/) was modeled after Alzforum to focus on schizophrenia research. SRF was built using the same software code as the original Alzforum site, and thus has an identical look, feel, and functionality to the previous version of Alzforum, before its 2013 re-launch. However, tools to rapidly create and customize such communities were not readily available and therefore the software cost to launch one such community could not be effectively amortized across others using a common software model.

To address this need, we decided to create a reusable platform to build biomedical web communities. We developed the Science Collaboration Framework (SCF) to provide the building blocks for these communities. An earlier version of the platform was developed primarily to publish scholarly articles in biomedicine that can be indexed by the National Library of Medicine (NLM) PubMed library (Das et al., 2009). Our newer version has been re-engineered to add social media and community features similar to those available in Alzforum. We also incorporated elements of Semantic Web technologies (Berners-Lee et al., 2001) to facilitate interoperability and interdisciplinary communications. Semantic Web is a technology developed by the World Wide Web Consortium (W3C), which aims to promote interoperability of Web content and creation of a “Web of Data” through the use of machine-readable Web pages. It relies heavily upon the used of agreed common vocabularies to describe objects and relationships in the Web. Biomedical science has developed a very rich set of such vocabularies or “ontologies” (over 300 vocabularies with over 5 million terms registered in the National Center for Biomedical Ontology at Stanford University Medical School). Use of ontologies such as these permits, among other benefits, resolution of the many synonym terms in biomedicine, to single common identifiers. Enabling Semantic Web technologies on our framework is meant as a step toward better integration with biomedical vocabularies and databases.

We chose the research area of chronic pain as the first use case. Chronic pain significantly impacts quality of life and is a substantial, growing, and unmet medical need worldwide. Although researchers have made great strides in understanding the underlying mechanisms and neurobiology of pain, few of these discoveries have been translated into new treatments. According to a recent report from the US Institutes of Medicine, chronic pain affects an estimated 100 million people in the US, and costs $600 billion annually in health care and lost productivity (National Research Council, 2011); the world-wide toll is unknown. For the most part, chronic pain conditions lack medications that are effective and well tolerated. One of the roadblocks to new treatments is a lack of communication and collaboration between basic, translational, and clinical researchers in the diverse scientific fields and clinical specialties that make up the pain research community. Thus, we developed an online open community, Pain Research Forum (PRF, http://painresearchforum.org), for pain researchers to freely exchange ideas and collectively elevate discussion of the causes of chronic pain and how that knowledge can be translated into new treatments and better care.

## Materials and methods

We have developed a reusable platform—SCF—to build science communities in focused biomedical areas. Previous versions of the platform included tools to publish scientific review articles following the NLM Document Type Definition (DTD), which can be indexed in PubMed (Das et al., 2009). The new release, used for PRF, includes a large number of additional community features, including means to publish news articles, forums, member profiles and various community and research resources. These features are described in the following sections.

### Architecture

The SCF is developed on an open-source content management system, Drupal 7^1^. Drupal is based on the PHP programming language and MySQL database running in the Linux/Apache web server environment. We chose Drupal because it is easily extensible and there are 30,000 registered Drupal developers continually contributing modules and enhancements to the core features^2^. We developed several custom content types and packaged them as features that can be installed and reused on any science community site. The graphic design (colors, fonts, etc.) is customized for each site using a theme layer (Kumar, 2012). The key content types available in the SCF are described in the next sections. The software is freely available upon request and a complete manual for editors to manage the site is under development.

### News articles and forums

The number of papers published in scientific journals continues to grow at a double-exponential rate (Hunter and Cohen, 2006) and it is becoming increasingly difficult for researchers to keep up with the literature. One way to address this problem is to publish news articles that summarize the research and provide context. We have developed a news feature that allows editors of the site to readily publish original news articles on emerging research. The News article has the following main fields: title, subhead, author, and body. The body text is composed in a WYSIWYG editor that allows flexible styling and the ability to add images. News items have references that are implemented as links to bibliographic listings of papers (described in section Papers of the Week). We wanted an easy method for researchers to find relevant news from a certain field, thus news items are categorized with terms from a pre-defined taxonomy. News articles can also be related to other news stories or papers, which appear in a block to the right of the article.

Discussion forums are important social media tools that enable interactions among researchers. Forums have fields similar to those of News. Forums can be Discussions of open research questions, structured Webinars or Journal Clubs. Videos and images can be embedded in Discussion, Webinars, or Journal Clubs. Site editors can specify related items for any Discussion, Webinar, or Journal Club.

Each News article and Forum can be commented on, bookmarked, watched, recommended or shared using social media tools.

### Papers of the week

Hundreds of papers are published weekly in PubMed for a given biomedical domain such as pain, and editor-curated weekly digests can help researchers stay abreast of the growing literature. Thus, we decided to create a module for curating and annotating papers downloaded from the NLM PubMed library. We use the previously developed PubMed module to import biomedical articles from PubMed using its Application Programming Interface (API) (Sayers, 2008). The Drupal biblio module^3^ is used to represent papers. We further developed the Journal Stream module that runs nightly queries using the software utility cron and imports the results in batches of 100 items. Complete documentation including a screencast and software for the Journal Stream module is available^4^.

**Figure 1:**
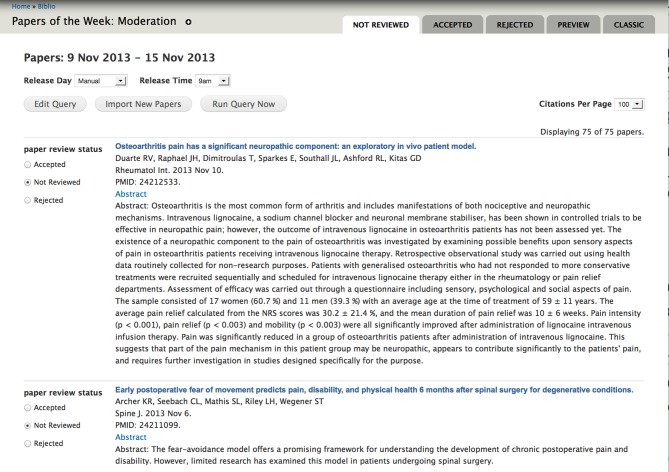
**Papers of the Week moderation queue**. Papers are imported nightly from NLM PubMed using a tailored query. Editors are presented with an easy-to-use interface to accept or reject the papers.

Each imported item is presented to the editors in a moderation queue and can then be “accepted” or “rejected.” A screen shot of the moderation queue is shown in Figure 1. The abstract of each paper is displayed so that editors can determine whether the paper should be accepted or rejected. Key papers can be selected as Editors' Picks, and editors can choose to highlight the paper with a few sentences. The accepted papers are published as weekly collections; the periodicity of posting the collections can be configured per site. Once the collection is published, each paper can be individually commented on, bookmarked, watched, recommended or shared using social media tools. Users may download paper citations into the EndNote reference management software using the Endnote XML format. Currently, only EndNote is available to PRF users, but the Drupal biblio module allows site administrators to enable the BibTeX format if desired, for import into various other reference management tools such as Mendeley, Reference Manager, or Papers.

The citation and Medline Subject Headings (MeSH) terms associated with each paper are automatically updated periodically, as new information is posted in PubMed.

### Members and registration

Members are the most important component of an online scientific community. We developed tools for members to join the site and publish their profile. While much of the content on the site is freely accessible without registration, only members can post comments on the site and have access to other members' profiles. The registration process starts with the members signing up online using a form. Research credentials including affiliation, position and research interest fields are required. The full name, email, city, and country are also required. Members agree to terms and conditions of membership on the site. The editor is notified when a member signs up and once the registration has been reviewed and approved, the new member receives an email informing them of the approval. Email authentication is required for membership activation and reminder emails are sent if members have not responded to the authentication request by 1 week after approval.

Members can publish detailed research profiles on the site, upload their biography, and have the opportunity to import a list of publications directly from PubMed. A member's contributions to the site are also listed on his or her profile page. Members can choose to allow other members to contact them via email functionality provided on the member profile page. Finally, members can subscribe to receive email alerts on new content by type (News, Webinars, Jobs, etc.).

### Community and research resources

We provide a variety of structured resources for members: Meetings and Events, Jobs, Funding Opportunities and Bulletin Board. Meetings and Events allows researchers to quickly find upcoming meetings of interest. These are listed automatically in reverse chronological order. Meetings can be linked to PRF Blogs and News stories on the event, allowing researchers to “catch-up” if they missed the actual event. Jobs provide networking between hiring institutions and applicants; Funding Opportunities highlights grants in the field and Bulletin Board is for posting *ad hoc* announcements. Together, these community resources provide content tailored to the professional needs of researchers in the pain field. All community resource items have social media tools and can be individually commented on, bookmarked, shared or recommended.

We also provide a variety of tools for creating and publishing research resources. Some are simply pages of information or collections of links to other useful resources. We also developed a structured database for genes associated with a disease or biological condition such as pain. Fields include data to fully describe the gene and details of data on variants associated with pain, with literature references. For studies using research models, the type of model is described, thus presenting a detailed overview of the research done to associate the gene with the disease. In the future, we would also like to create other resources, such as a drugs database that would serve as a central repository for information on new drugs in development and associated clinical trials.

### Social media tools

Social media tools are important for online collaboration. We developed or customized a large number of social media tools and incorporated them in our framework. Members can comment on or invite others to comment, share, bookmark, and recommend most content throughout the site. All content on PRF can be emailed and shared on all the popular social network tools (Facebook, Twitter, etc.) by using standard “email” and “share” modules present on every page. RSS news and Twitter feeds are available as well as an email newsletter.

To accommodate the needs of our scientific community, we made a large number of enhancements to the comment feature in Drupal. Scientific commentaries often have attachments or figures, so we developed capabilities for attaching images or documents. The comments can be formatted with a WYSIWYG editor and can be associated with more than one content item if applicable.

### Web site use and tracking

Websites are tracked using Google Analytics^5^, which provides extensive data on how users interact with the site. We analyze data on number of visits, unique visitors, total pageviews, and views of individual pages. We also look at selected demographic data (country and city of origin), system information, and source of traffic to the site.

### Search and semantic web

Search is implemented using the open-source enterprise Apache Lucene Solr^6^ search platform. We also implemented section-specific searches. Search results can be sorted by date, relevance, number of comments or the date of the last comment. The number of search results per page can be configured by the user. Search results can be filtered using facets. The content type, date, categories etc. are presented as facets.

Semantic Web technologies enable publication of structured documents that can be processed by machines, thus allowing interoperability with the Web of Data (Berners-Lee et al., 2001). We use the Drupal Resource Description Framework Modules (RDF) modules (Corlosquet et al., 2009) to publish RDF of News and Forums. The RDF is indexed and stored in a SPARQL endpoint using the PHP ARC2 libraries^7^. The Dublin Core (Weibel et al., 1998) and Semantically-Interlinked Online Communities (SIOC) (Breslin et al., 2006) ontologies are used to express the RDF. SPARQL queries enable us to perform flexible queries and integrate with other knowledge repositories. Thus, incorporation of Semantic Web technologies in the SCF platform will allow us to network additional online communities built with SCF and identify relevant information across multiple sites.

## Results

### Pain research forum

**Figure 2:**
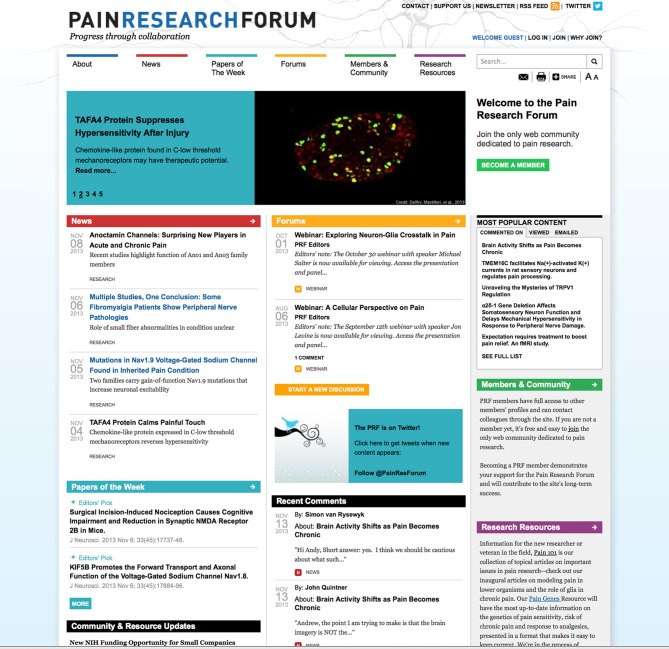
**Pain Research Forum**. Screenshot of home page for Pain Research Forum (http://www.painresearchforum.org) for anonymous users (not logged-in).

The SCF platform, originally used for publishing scholarly articles for StemBook (http://stembook.org), was reengineered to create an online community of chronic pain researchers. The goal was to accelerate pain research by enabling free discussion and faster sharing of information between academia, industry, and the clinic, to foster new collaborations and to raise interest in pain research among the wider community of neuroscientists and clinicians. The PRF^8^ was launched in June 2011; a screenshot of the home page is shown in Figure 2. As of December 2013, PRF has attracted ~1400 registered members and has published more than 150 News stories and 25 Discussion forums, facilitated five Webinars and published four Journal Clubs features. There are more than 200 member-authored comments on News, Papers, and other content. Papers of the Week are published every Friday and 2–6 papers are highlighted each week as Editors' Picks. Curated and frequently updated lists of Meetings and Events, Jobs, Funding Opportunities, and Bulletin Board items are posted. The site editors actively curate and maintain several research resources. All content is licensed for reuse under Creative Commons license BY-ND-NC^9^.

### News

**Figure 3:**
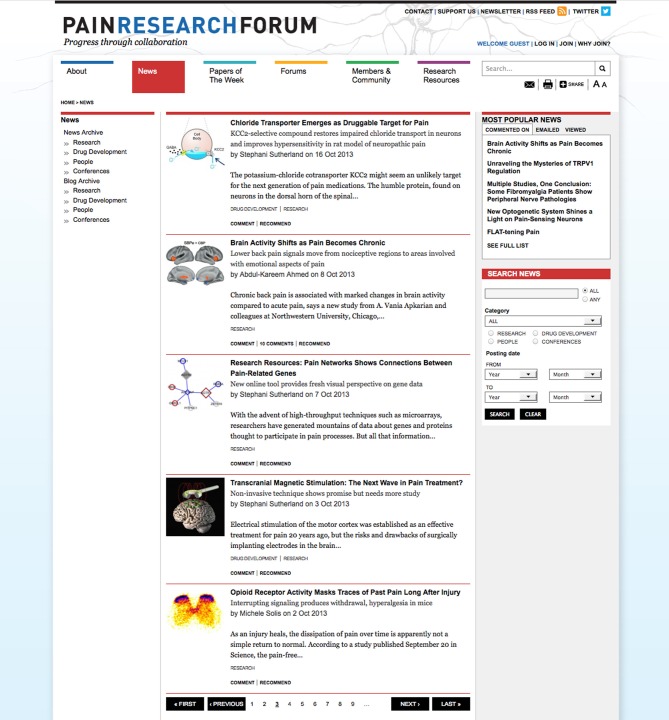
**News Section in PRF**. Screenshot of news section in Pain Research Forum (http://www.painresearchforum.org/news). Five stories are listed per page, social media tools are available for each story. News stories can be filtered using categories on left. Most popular items are highlighted on the right.

PRF publishes 1–2 news stories each week; a screen shot of the News section is shown in Figure 3. News stories are categorized as “Research,” “Drug Development,” “People” or “Conferences.” PRF's news coverage helps researchers stay abreast of the latest findings in the field. For example, PRF was one of the first media outlets to publish a news story about a study of how “high-dose opioid reverses synaptic potentiation in the spinal cord in rats” (Talkington, 2012). The research paper was published in Science on January 13, 2012 and the PRF news story came out 3 days later. Four prominent pain researchers presented their opinions on the work in the form of comments to the story.

**Figure 4:**
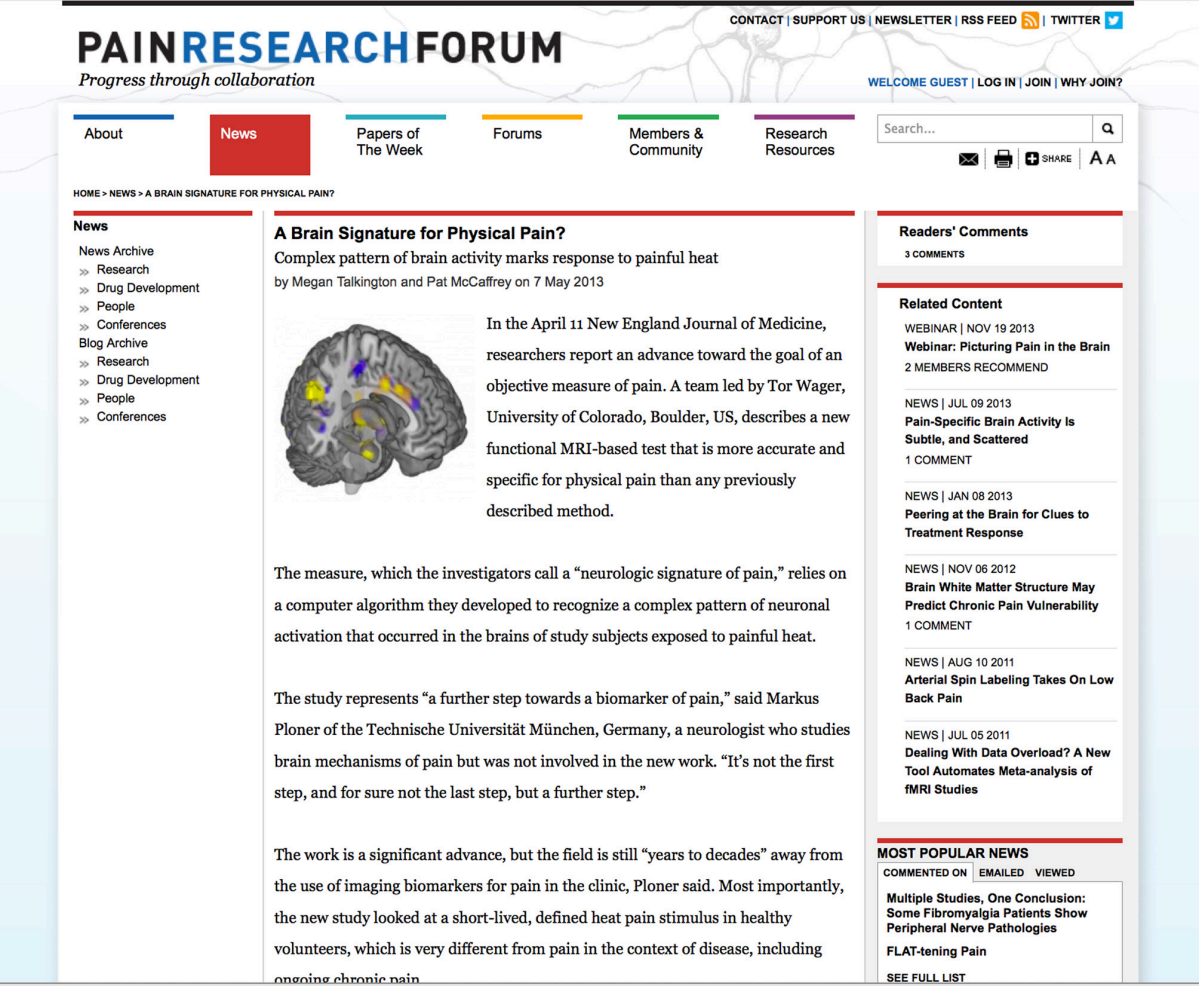
**News story on brain imaging**. Screenshot of news story on brain imaging study (http://www.painresearchforum.org/news/27192-brain-signature-physical-pain). Study was led by Tor Wager, University of Colorado, Boulder, US and describes a new functional MRI-based test for measuring pain. Related stories are listed on a block on the right. The article has 3 comments.

**Figure 5:**
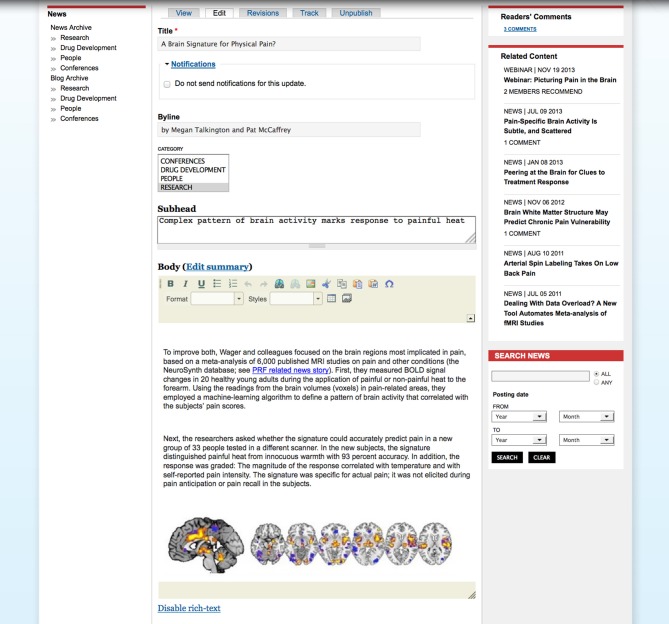
**Forms used by editors for a News story**. Screenshot of forms used by the editors to create and publish a news story. A form is available for each field and the body is composed using a WYSIWYG editor.

Often several related stories are published on an individual topic, and SCF is engineered to allow integration of this material. For instance, PRF recently covered three high-profile brain-imaging studies (Ahmed, 2013; Talkington, 2013; Talkington and McCaffrey, 2013). A screen shot of one of the stories is shown in Figure 4. The forms used to create and publish the story are shown in Figure 5. Related stories are listed in a block on the right. In addition, PRF conducted a webinar in December 2013, featuring one of the principal investigators on the brain imaging papers, along with several panelists including authors of the other papers mentioned in the news coverage. The webinar is also listed as related content to the news story. By hyperlinking, using the References function, cross-posting comments on both news stories and papers, and using the Related Content feature, PRF is able to provide a contextualized, intelligent overview of fast-moving developments in this corner of the larger field. Social media tools such as Twitter feeds and newsletters disseminate the information quickly and effectively to members.

Brain imaging in the context of understanding and detecting pain is a popular but controversial topic, and the three stories cited above (Ahmed, 2013; Talkington, 2013; Talkington and McCaffrey, 2013) elicited several comments from PRF members. It is significant that many of the researchers commenting on PRF are junior people, including graduate students or postdoctoral fellows. Often the study authors participate in the discussion: for example, on the news story by Talkington and McCaffrey (2013), the study authors responded to two previous comments from researchers not involved with the study. Thus, the commenting feature on PRF news stories serves a function similar to the “letters to the editor” sections of journals, with key differences: it is faster, has a lower barrier to entry, and welcomes contributions from junior researchers.

Currently, the most accessed news story is one that discusses the new research on the use of antibiotics to relieve some forms of chronic lower back pain (Morton, 2013). The PRF news story covers two published research studies: one suggests that pain may be caused by a low-grade bacterial infection in the discs and the other finds that antibiotics can effectively treat the pain and prevent further tissue degeneration. Both studies have implications for patients with long-standing low back pain, and they elicited a lot of attention and controversy including in the popular press. In a Google search for the query “antibiotics for back pain,” the PRF news story is the number one hit. This shows that PRF news stories can be highly ranked in Google searches for general pain terms, giving many readers access to the site. This high ranking may contribute to the unusually high number of page views for this article, which are three times more than the second most viewed news article.

### Forums

**Figure 6:**
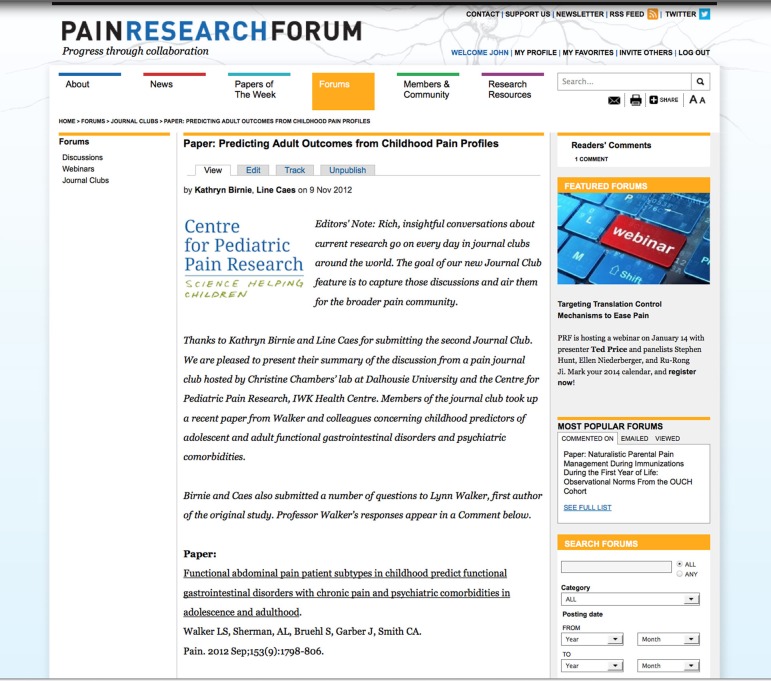
**Journal Club on recently published paper**. Screenshot of journal club featuring a recently published paper in the journal PAIN (http://www.painresearchforum.org/forums/journal-club/21586-predicting-adult-outcomes-childhood-pain-profiles). Featured forums are highlighted in a block on the right.

The PRF Forums category includes Discussions, Webinars, and Journal Clubs. PRF editors have moderated several online discussions. The most-accessed Discussion is a debate on how the human brain processes stimuli, initiated by a well known pain researcher and PRF Science Advisory Board member (Basbaum, 2011). A Discussion on the challenges associated with translating pain research discoveries into clinical developments, presented by another researcher and science advisor, attracted many follow-on comments (Mogil, 2011). In 2013, PRF conducted five Webinars, each typically attracting ~150 registered attendees, plus an unknown number of additional viewers who watched the event in groups under one registration. Each Webinar is conducted online using a webinar-hosting service and a recording is subsequently posted to PRF with a written introduction. This archives the presentation for future viewing and enables an ongoing, online conversation beyond the duration of the actual presentation. The Journal Club is a venue for disseminating the results of discussions that occur in individual lab groups about recently published scientific articles. For example, a graduate student and postdoctoral fellow studying pediatric pain presented a journal club at their institution on Walker et al. (2012; Birnie and Caes, 2012). They then wrote for PRF a brief synopsis of the study and the discussion that took place in their meeting. They also posed questions to the author of the original paper, who responded with her own online comment. A screenshot of the Journal Club is shown in Figure 6.

### Papers of the week

**Figure 7:**
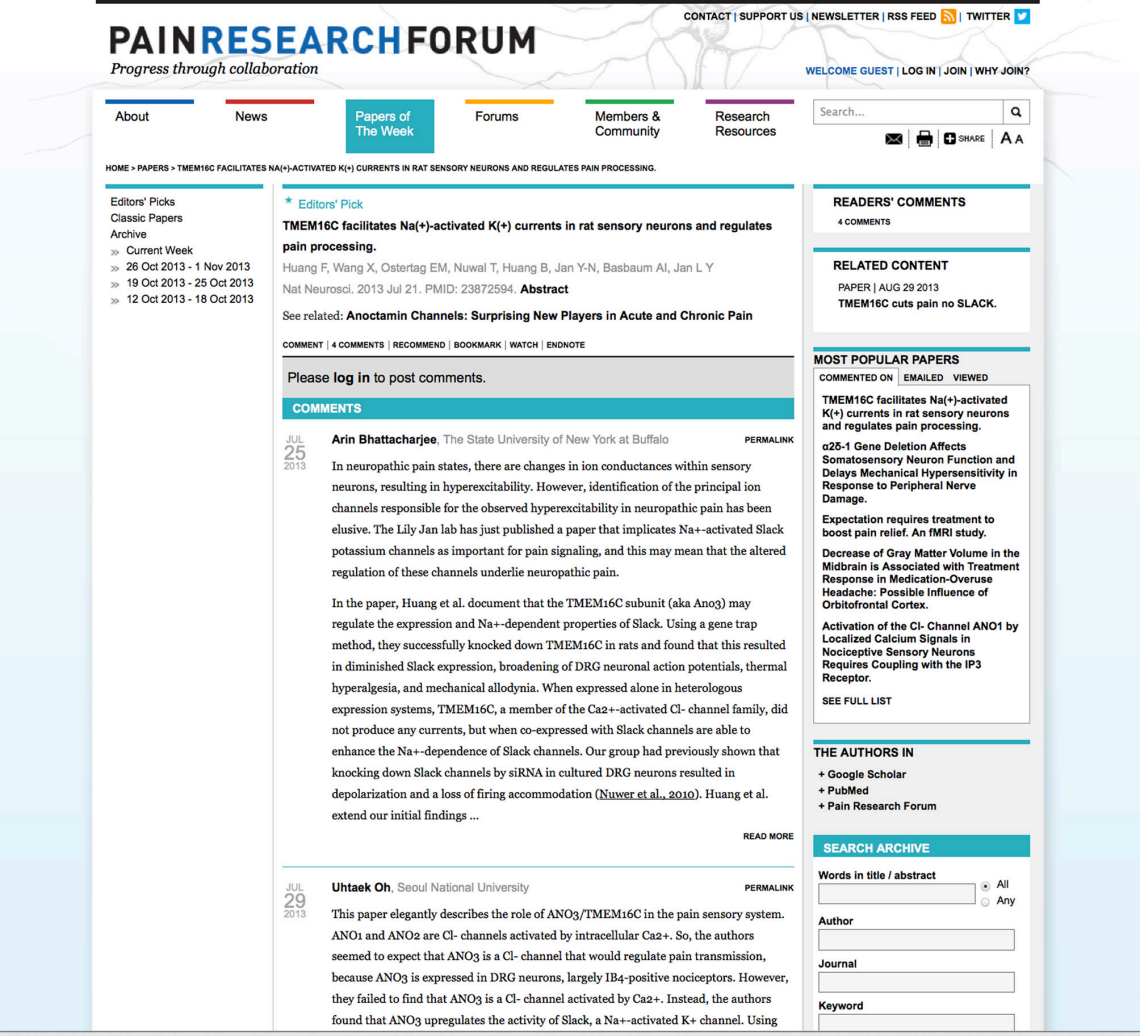
**Sample Paper from Papers of the Week**. Screenshot of paper published in Papers of the Week (http://www.painresearchforum.org/papers/29791-tmem16c-facilitates-na-activated-k-currents-rat-sensory-neurons-and-regulates-pain). Related paper is listed on the right. Section specific search is also available.

Papers of the Week are published every Friday and provide a curated digest of recent and noteworthy pain-relevant articles published in academic journals. For example, for the November 2–8, 2013 collection^10^, 61 papers were identified and listed. Two were further highlighted as Editors' Picks. Highlighted papers are often commented on by PRF-registered members, sometimes resulting in vibrant back-and-forth discussions between the authors and other PRF members^11^. Related papers are listed in a block on the right as shown in Figure 7; a news story on the paper is listed under the paper citation. Papers of the Week are archived as weekly lists, and the database of all papers can be searched with a detailed section-specific search. Members can also search for other papers using links provided on the paper page to Google Scholar or PubMed.

### PRF membership and usage statistics

**Figure 8:**
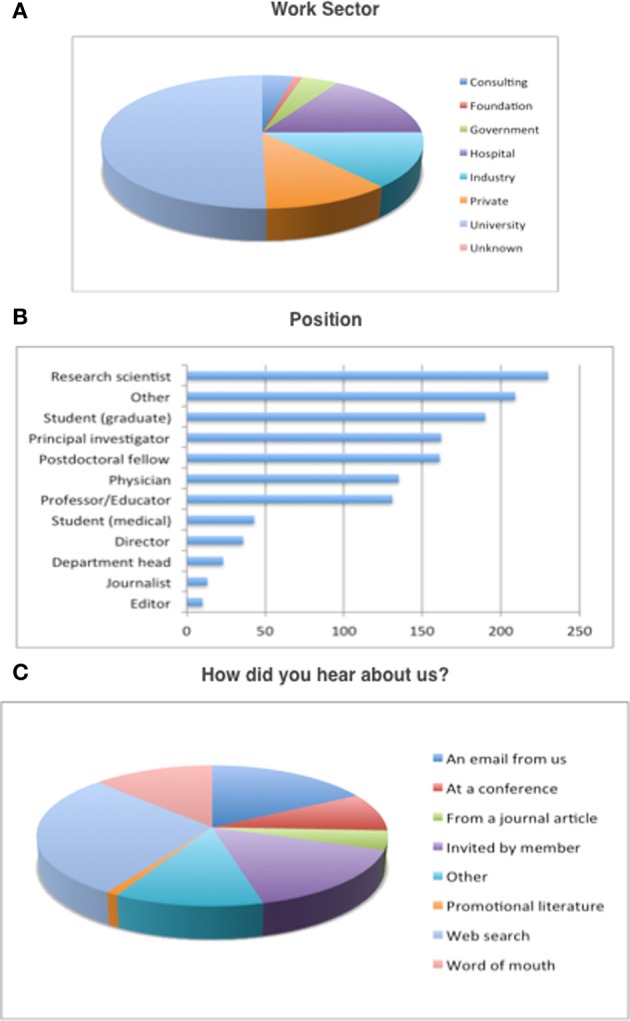
**Membership Statistics. (A)** Shows the distribution of work sectors, **(B)** shows the different position of members, and **(C)** depicts the how different members heard about PRF.

As of December 17, 2013, PRF has just over 1400 registered members, of whom 1143 have published profiles in the member directory. Member demographics are shown in Figure 8. A variety of professionals sectors are represented including universities, hospitals, industry, government, and non-profit foundations, with the majority of members coming from academia (Figure 8A). Most members are research scientists and academicians including graduate students and postdoctoral fellows (Figure 8B). Two-thirds (67%) of PRF members have an advanced degree, 41% have earned a PhD and 20% have an MD or DDS.

Although promotional efforts—emails, conference attendance, printed literature, etc.—have been responsible for attracting many members (Figure 8C), more than half of members found PRF either via a Web search, word of mouth or were directly invited to join by an existing member. Social media tools played an important role in recruiting members (“Other” Category).

We found that a significant proportion (about one-third) of newly registered members failed to respond to the email validation message, which asked them to click a link and return to the site to complete the registration process. We installed a module to automatically send reminder emails to these new members, and have recovered about half of the non-responders.

Google Analytics shows that in October 2013, PRF had ~8000 unique visitors, ~11,000 visits and over 25,000 page views. PRF has visitors from all over the world, with the majority from the USA, UK, Canada, and Australia. The most popular browsers are Chrome, Safari, and Firefox. The Papers of the Week and News are the most popular sections; popular community pages include Jobs and Meetings & Events. Some of the news stories mentioned above are among the top pages visited on the site.

### Community and research resources

PRF lists community and research resources of interest to the community, and the software automatically highlights new listings on the home page and section landing pages. Currently users can access information on more than 30 upcoming meetings and read coverage of past meetings. Job postings^12^, funding opportunities^13^, and bulletin board^14^ items are posted. In addition, several research resources are also provided including a curated pain gene resource consisting of about 25 genes that are associated with pain, according to peer reviewed, published studies, and a collection of “Pain 101” articles covering basic questions in pain research. A collection of useful links is planned.

### Search and semantic web

**Figure 9:**
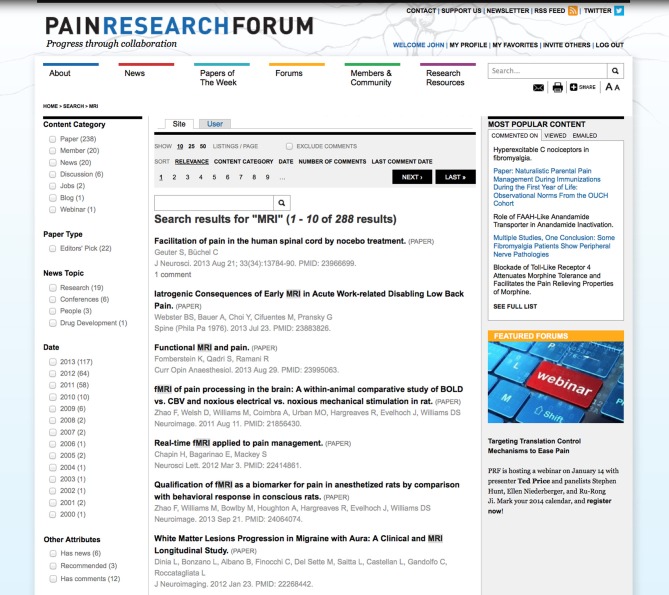
**Search Results**. This figure displays the search results for the term MRI. The search results can be filtered using various facets on the left and sorted by content type, date, number of comments or date of last comment.

We have implemented a general as well as section-specific search for PRF using the Apace Solr module as described in the Methods section. The search results can be further filtered by content type, date, news topic or whether an item is recommended or has comments (Figure 9). The search term is highlighted in the results and users have the option to sort the results by type, date, number of comments or last comment date.

We have created Linked data and RDF for News, Forums, and Papers using the Drupal RDF modules along with the SIOC and DC ontologies. A sample RDF^15^ illustrates the use of these ontologies to describe the News article. The RDF is indexed using the ARC2 PHP libraries and is available at http://www.painresearchforum.org/sparql. The SPARQL endpoint allows us to perform flexible queries such as “all News articles published with greater than 2 comments” (see Table 1). In the future, we could perform federated queries across endpoints from multiple communities.

**Table 1:** **Example SPARQL Query**.

PREFIX schema: <http://schema.org/>,
PREFIX sioc: <http://rdfs.org/sioc/ns#>
SELECT ?post ?title ?replies
WHERE {
?post a schema:NewsArticle;
schema:name ?title;
sioc:num_replies ?replies.
FILTER ($replies > 2)
}
ORDER BY DESC(?replies)

*Query to find news articles with > 2 comments*.

## Discussion

Our reusable platform, SCF, was successfully deployed to create a vibrant online community of chronic pain researchers: PRF. In a little over 2 years, PRF has attracted a large community of registered users and contributors including members from academia, industry, government, and non-profit organizations. Scientists engaged in laboratory research, clinicians, students, and fellows are all represented. PRF is a network of investigators from various sectors and disciplines and a venue for discussing, critiquing, and advancing pain research. The response to PRF in terms of member registrations and site use indicates a pent-up demand for such online communities that provide researchers in disease-circumscribed fields of biomedical research with news, forums, and resources that are most relevant to them in one place.

In our experience, editorial involvement is crucial to keep the site active and vibrant with user interactions. Reporting news, moderating discussions, soliciting comments and producing webinars and journal club pieces is labor intensive but necessary to maintain high quality interactions within the user community. On PRF, this is accomplished by a staff of professional editors and writers with backgrounds in research and neurobiology, whose primary responsibility is to create and moderate content. Assembling an active and engaged Scientific Advisory Board made up of leading researchers and clinicians from a variety of disciplines is also important to ensure the highest quality content, provide community outreach and promote community involvement.

PRF is publicized in the pain research community by several avenues. The launch was announced with a press release and with a direct email to several thousand pain researchers, identified through meeting rosters, publications, and a existing pain research listserve. In addition, fliers were distributed at pain meetings and neuroscience meetings, including 7000 postcards placed in meeting bags at the most recent World Congress on Pain in Milan (2012), the largest gathering of pain researchers in the world. Several pain professional groups promote the site to their members on their websites or in member newsletters. PRF editors have given talks and posters at pain conferences. The PRF science advisors promote the site to their colleagues using slides and other materials provided by PRF. The site is also marketed through word of mouth, a monthly email newsletter, and RSS and Twitter (@PainResForum) feeds.

One barrier to progress in research is the reticence of researchers to divulge unpublished or other preliminary work, or to publicly criticize the work of others. Web communities like PRF provide a new model of open communication that will help change this culture and promote faster and freer information exchange. A barrier to achieving more researcher involvement in and contributions to communities like PRF is the lack of incentives for scientists to contribute comments or other materials that do not add to their official publication record. In the future, sites like PRF should aim to provide incentives for contribution, for example by indexing content on PubMed or by arranging to provide continuing medical education (CME) credits for physicians who contribute.

All content on PRF is provided free of charge to the research community, and funding of shared resources like PRF is an ongoing challenge. To attract donors and other sponsors of the site we must continually demonstrate both scientific credibility in all of the content presented, and constant, lively and intellectually valuable interactions. Consistent outreach to and education of potential funders in the philanthropic area, professional societies, relevant pharmaceutical and biotech companies and the academic sector is also required. At the same time, editorial independence from sponsors must be strictly maintained. PRF does not accept paid advertisements and does not intend to do so.

In terms of technology, the SCF platform consists of a comprehensive set of building blocks for an online community. We have effectively used the SCF platform to create other communities: the Multiple Sclerosis Discovery Forum (msdiscovery.org) and StemBook (stembook.org). The platform incorporates elements of Semantic Web technologies, which have the potential to accelerate the pace of inter-disciplinary research by defining a common language and improving interoperability between various resources on the Web (Hendler, 2003). In the future, we plan to leverage these Semantic Web technologies to enable us to do cross-site queries and find relevant information on other sites. Interoperability between these multiple communities involved in neurobiology and neurology research will, we hope, identify common biological mechanisms behind complex neurological diseases and accelerate translation of science to new treatments.

### Conflict of interest statement

The authors declare that the research was conducted in the absence of any commercial or financial relationships that could be construed as a potential conflict of interest.

## Footnotes

^1^http://www.drupal.org

^2^http://en.wikipedia.org/wiki/Drupal

^3^https://drupal.org/project/biblio

^4^http://scf.github.io/journalstream/

^5^http://www.google.com/analytics/

^6^http://lucene.apache.org/solr/

^7^https://github.com/semsol/arc2

^8^http://www.painresearchforum.org/

^9^http://creativecommons.org/licenses/by-nc-nd/3.0/

^10^http://www.painresearchforum.org/node/33603

^11^http://www.painresearchforum.org/papers/22720-activation-5-ht2a-receptors-upregulates-function-neuronal-k-cl-cotransporter-kcc2

^12^http://www.painresearchforum.org/members/jobs

^13^http://www.painresearchforum.org/members/funding-opportunities

^14^http://www.painresearchforum.org/members/bulletin-board

^15^http://www.painresearchforum.org/node/34289.rdf
